# Case Report- Prevention strategies for permanent tracheostomy tube migration into the left main bronchus in tracheostomized patients

**DOI:** 10.12688/f1000research.163217.1

**Published:** 2025-04-01

**Authors:** Said Khallikane, Amine Bentahar, Monsif Salek, Ayoub Belhadj, Younès Aissaoui

**Affiliations:** 1 Faculty of Medicine and Pharmacy, Cadi Ayyad University, Marrakech, 40000, Morocco; 2 Anesthesiology-ICU-Emergency Department, Avicenna Military Hospital, Marrakech, 40000, Morocco; 3 Faculty of Medicine, Pharmacy and Dentistry, Fes , Kingdom of Morocco., Sidi Mohamed Ben Abdellah University, Kingdom of Morocco, 30000, Morocco; 4 Imaging and Interventional Radiologist, Diagnostic and Interventional Radiology Department., Moulay Ismail Military Hospital, Meknes, 50000, Morocco

**Keywords:** Tracheostomy tube migration, airway obstruction, bronchoscopy, respiratory distress, supraglottic carcinoma, spontaneous ventilation anaesthesia.

## Abstract

Tracheostomy tube migration is a rare but potentially life-threatening complication. We present the case of a 66-year-old male with chronic obstructive pulmonary disease, dual-chamber pacemaker implantation for chronic ischemic cardiomyopathy, and supraglottic squamous cell carcinoma. The patient developed respiratory distress due to intrabronchial migration of the tracheostomy tube. Initial evaluation revealed significant respiratory effort, inspiratory stridor, and an absent tube at the stoma site. Imaging and bronchoscopy confirmed the tube’s presence in the left main bronchus with associated mucosal inflammation. The patient underwent bronchoscopic-guided tube removal and successful repositioning of a cuffed tracheostomy tube under spontaneous ventilation anaesthesia. Following stabilization, he was discharged with plans for a permanent tracheostomy. Tracheostomy tube migration presents a significant diagnostic and therapeutic challenge, particularly given the scarcity of studies involving adults with this complication. This case highlights the importance of early recognition, prompt imaging—especially bronchoscopy—and tailored management strategies, while emphasizing the active involvement of the patient and family in the care pathway. It also underscores the necessity for vigilant monitoring to prevent severe, potentially fatal, complications.

## Introduction

Tracheostomy is a common surgical procedure performed on patients requiring prolonged mechanical ventilation, those experiencing acute or chronic upper airway obstruction, or those needing airway protection. ^1^ Temporary tracheostomies are performed on patients undergoing ventilatory weaning, whereas permanent tracheostomies are indicated for patients who require a continuously open airway. ^1^ However, complications such as tube malposition or migration can lead to significant morbidity and mortality. ^2^ Migration into the main bronchus is particularly dangerous, as it can result in airway obstruction, hypoxia, and lung collapse. ^2^ When combined with underlying cardiac conditions, the risk of hemodynamic instability increases. Documented complications include infection, hypoxemia, and granulation tissue formation, underscoring the importance of proper tube positioning to prevent severe health issues. In some cases, patients with no prior complications may experience airway obstruction when the tracheostomy tube is found in the left main bronchus or other parts of the tracheobronchial tree, particularly in the presence of respiratory distress, cardiopulmonary arrest, or neurological deterioration. ^1^

Tracheostomy tube migration may result from tube movement; however, studies specifically addressing this phenomenon remain scarce and lack comprehensive evaluation, particularly in the adult population. ^3, 4^ It is imperative to establish precise mechanisms and standardized guidelines to prevent such complications in tracheostomized patients. In this study, we present a case of a tracheostomized patient with an implantable pacemaker who developed airway obstruction due to tracheostomy tube migration into the left main bronchus. This case illustrates the potential mechanisms underlying tracheostomy tube migration and analyzes similar cases from the literature, encouraging the exchange of experiences regarding the prevention and diagnosis of this significant clinical challenge.

## Case history and examination

A 66-year-old man with a history of history of chronic smoking for 35 years (two packs per year, not weaned), coronary artery disease with a prior NSTEMI requiring stent placement in the proximal left anterior descending artery, and an implantable pacemaker for cardiac arrhythmia currently stable on maintenance therapy. He also had chronic obstructive pulmonary disease (COPD) managed with short- and long-acting bronchodilators, high-dose inhaled corticosteroids (fluticasone 250 μg three times daily), and tiotropium bromide (18 μg twice daily). His respiratory insufficiency was aggravated by progressive-onset stridor and inspiratory distress over the past six months. A cervicothoracic CT scan and nasofibroscopy confirmed a diagnosis of supraglottic squamous cell carcinoma classified as T4a N2 M0, characterized by thyroid cartilage invasion and bilateral metastatic lymphadenopathy measuring 3 to 5 cm, with histopathological analysis confirming a moderately differentiated (grade 2) carcinoma. His surgical history included endovascular repair of an ascending aortic aneurysm two years earlier, mechanical aortic valve replacement for a bicuspid valve complicated by endocarditis, followed by debridement, a homo-Valsalva graft, and aortic root graft repair. Due to airway compromise caused by the supraglottic carcinoma, a tracheostomy was performed. He was admitted to the emergency department with progressive respiratory distress, dyspnea, tachypnea, and mild desaturation in room air (SpO2 at 92%), associated with significant respiratory effort involving the use of accessory respiratory muscles. The patient had been on long-term tracheostomy support following prolonged ventilation due to an unresectable malignant supraglottic stenotic process.

On clinical examination at the emergency department, the patient appeared fatigued, with neck fullness and decreased bilateral breath sounds, along with expiratory wheezing. The permanent tracheostomy tube was absent, with an inflamed stoma site and purulent green secretions. There were no signs of right or left heart failure. Pulmonary auscultation revealed diffuse inspiratory stridor and poor air entry in both lungs, particularly on the left side, without pulmonary crackles. His vital signs showed a temperature of 37°C, heart rate of 100 bpm Independent of the pacemaker, respiratory rate of 33, spontaneous ventilation with oxygen administered through the tracheostomy stoma, signs of respiratory effort, and an oxygen saturation of 98% on 10 L/min oxygen therapy.

**Figure 1.**
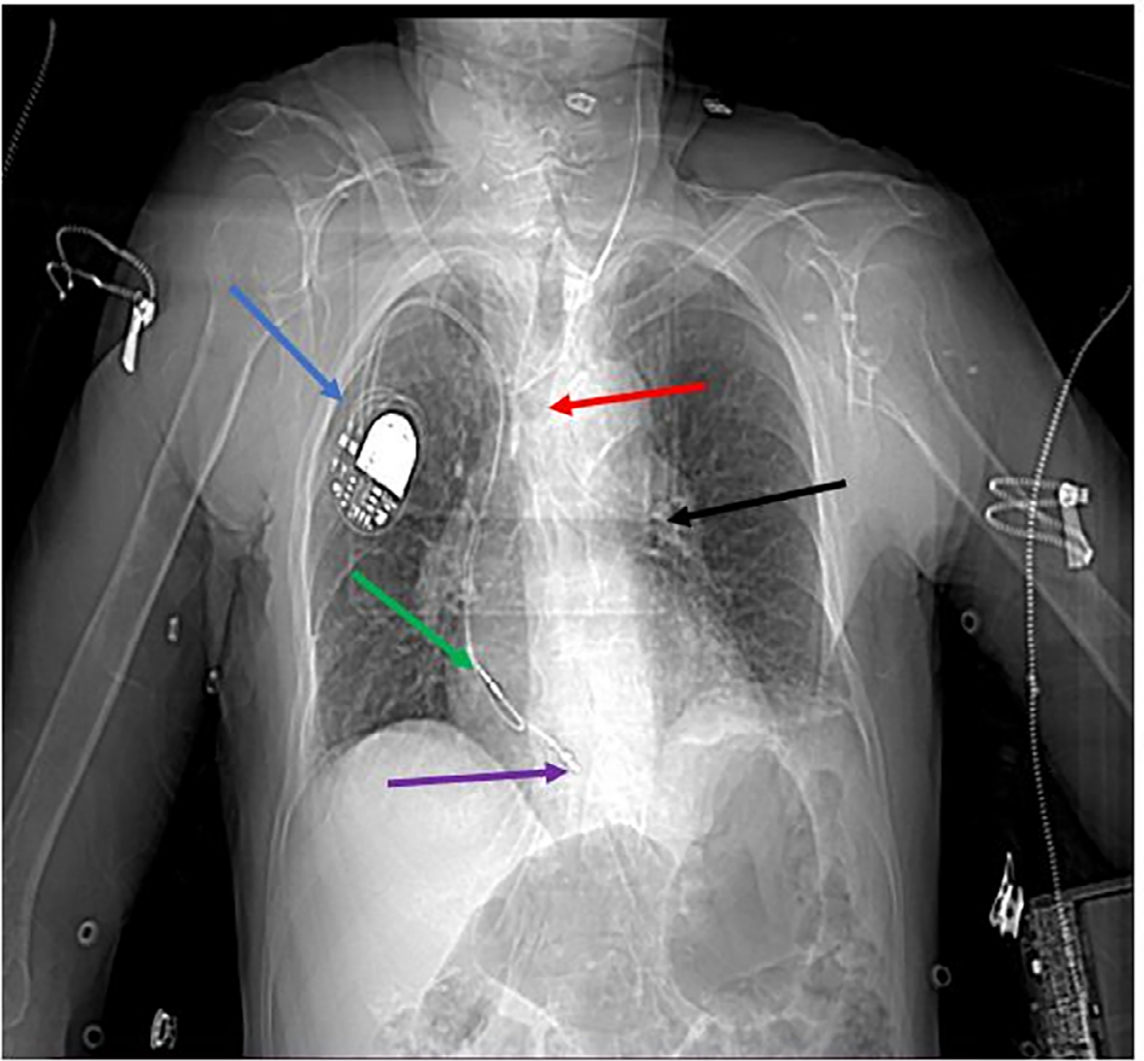
Chest X-ray showing pacemaker position (blue arrow), lead Pathway (green/purple arrows), tracheal Deviation (red arrow), and Foreign Body (black arrow).

Chest X-ray showed a tracheostomy tube positioned horizontally below the level of the carina, within the left mainstem bronchus ( Figure 1).

**Figure 2.**
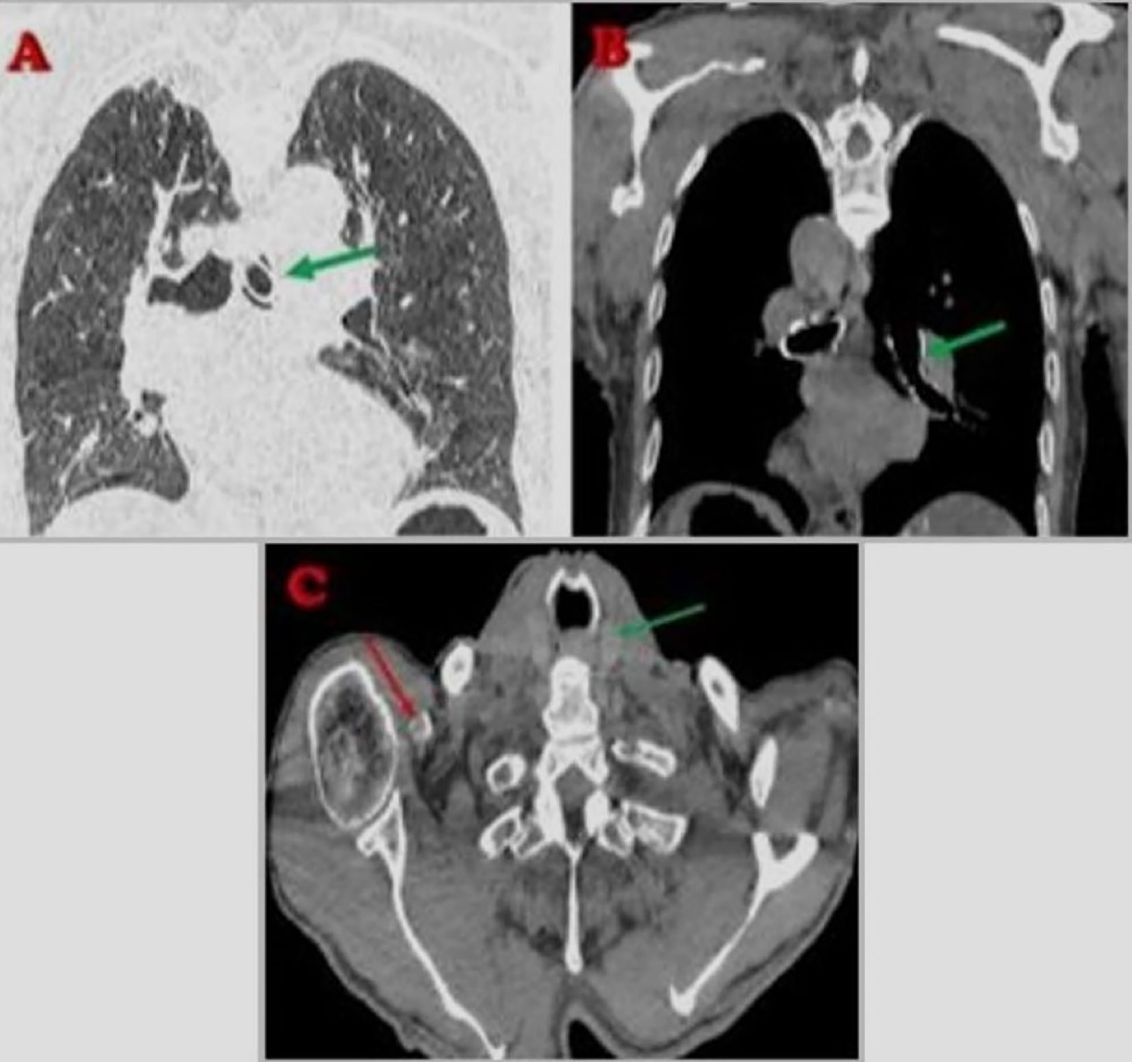
CT-Scan showing tracheostomy tube misplacement (green arrow), mediastinal shift, and pacemaker position (red arrow).

**Figure 3.**
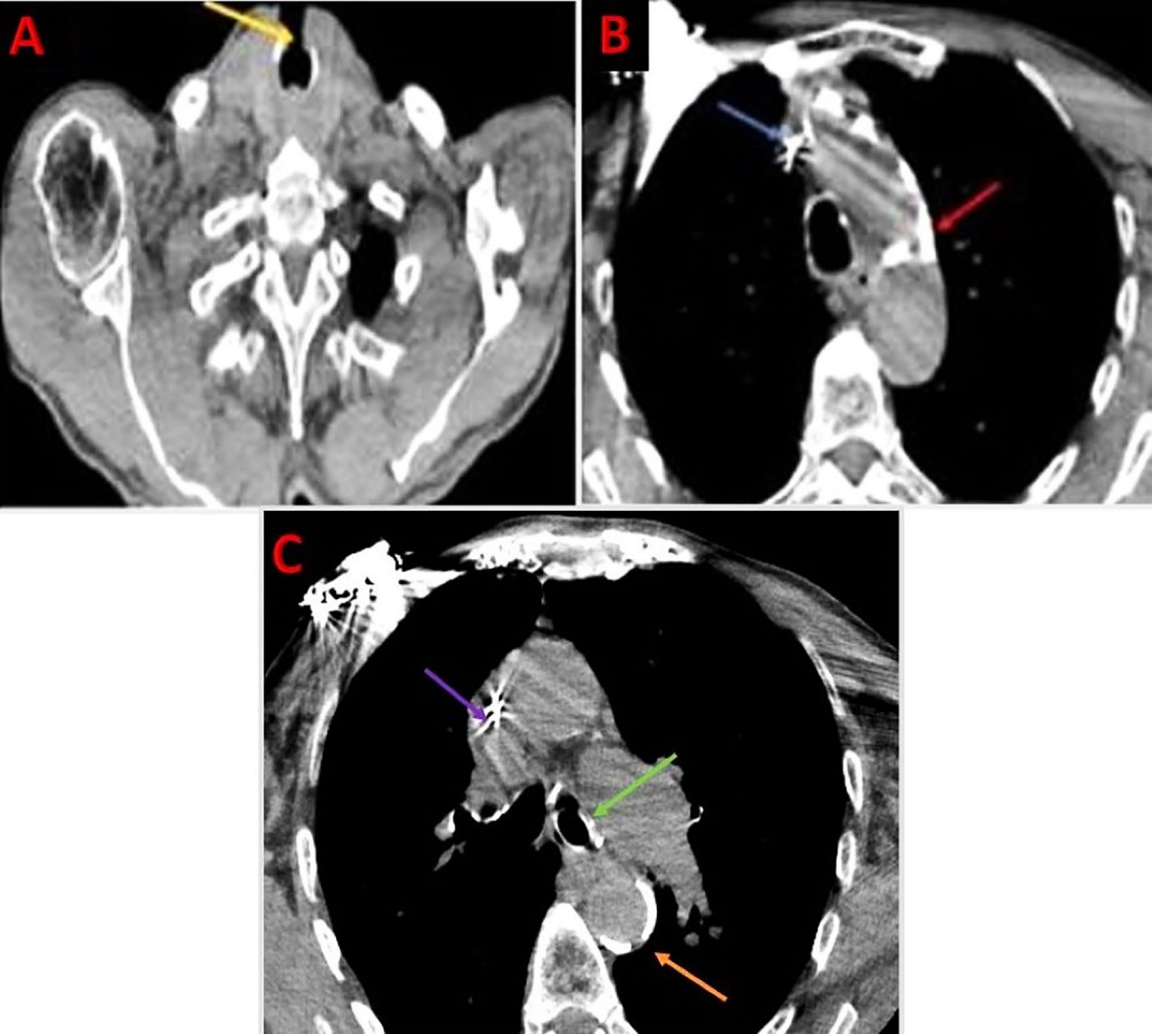
CT-Scan showing tracheostomy orifice (yellow arrow), misplaced Tube (green arrow), and vascular Calcifications (red/orange arrows).

A cervicothoracic CT scan revealed supraglottic malignancy. Additionally, cervical and mediastinal lymphadenopathy was noted. The tracheostomy tube was mispositioned, extending into the left main bronchus and causing partial airway obstruction. An implantable pacemaker was present, with its leads appropriately positioned ( Figures 2, 3).

**Figure 4.**
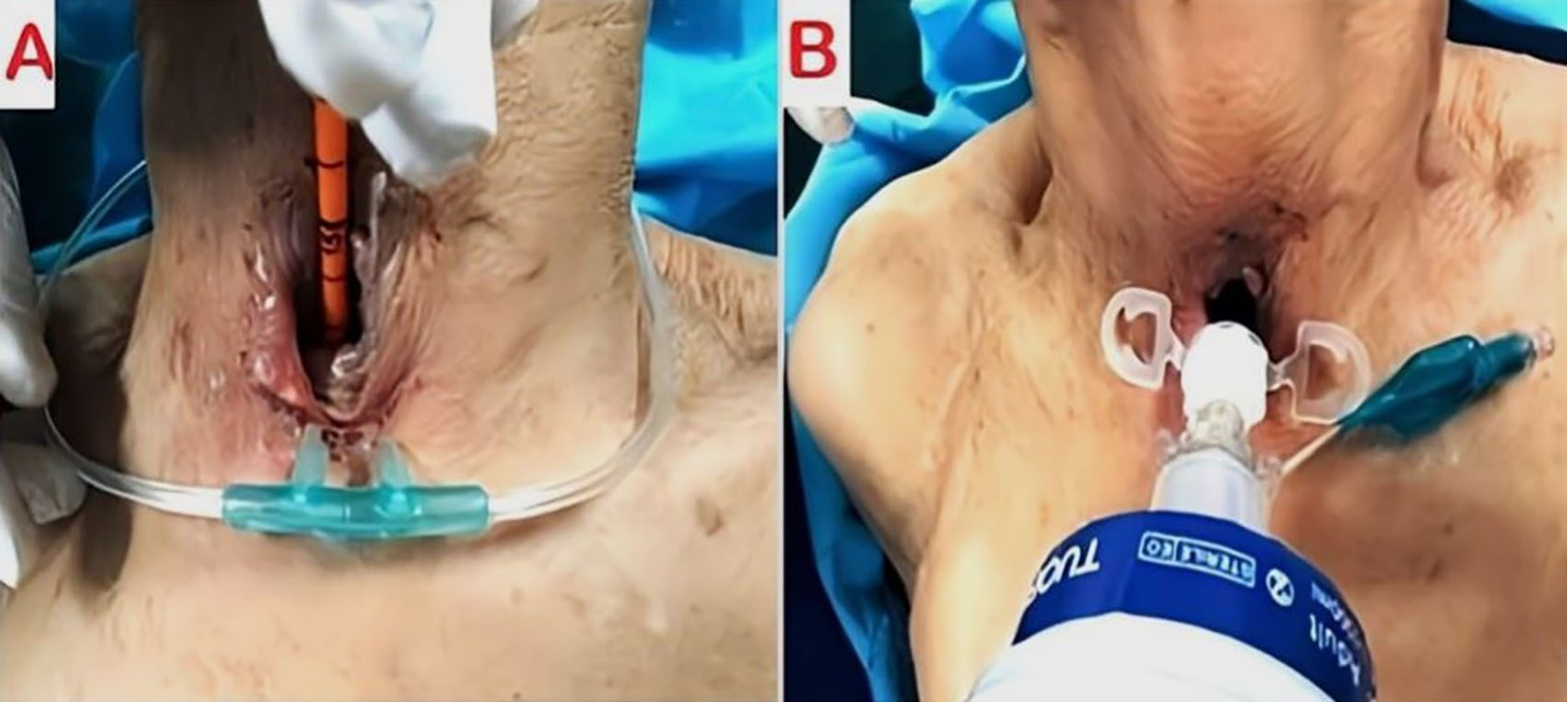
Intraoperative placement of cuffed tracheostomy tube (A) using guidewire (B) for airway management.

**Figure 5.**
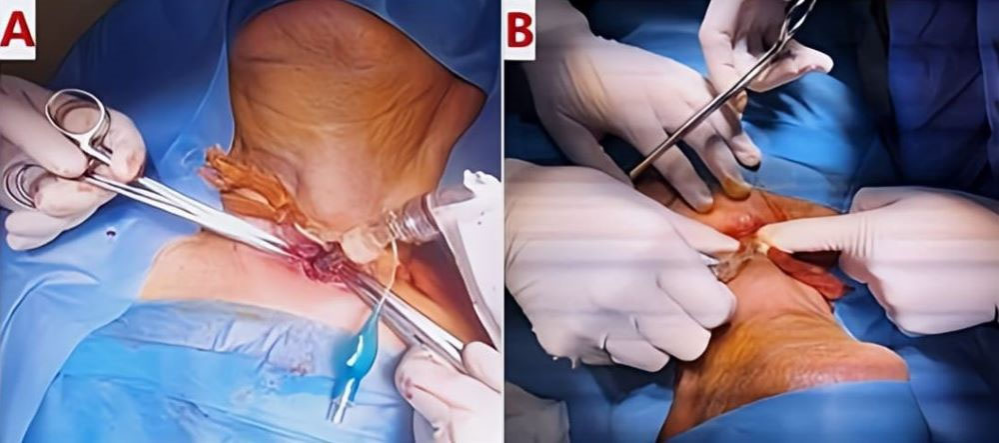
Intraoperative tracheostomy tube placement with airway dilation and granulation tissue excision (A, B).

**Figure 6.**
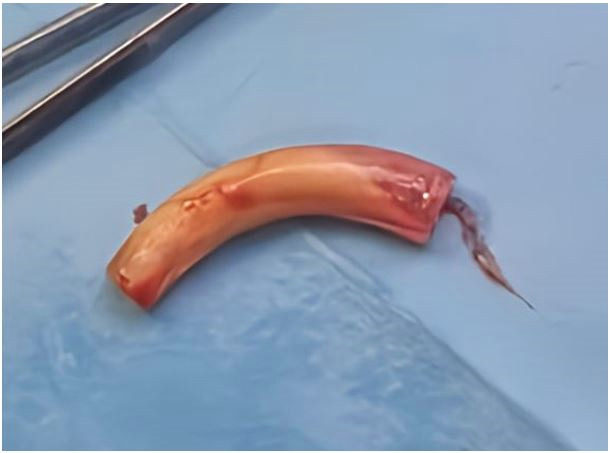
Extracted tracheostomy tube from left main bronchus showing discoloration and mucosal Debris.

The patient underwent urgent bedside bronchoscopy through the tracheal stoma, which confirmed that the tracheostomy tube had migrated into the left main bronchus, with signs of mucosal inflammation. Nasofibroscopy revealed an inflammatory appearance and a non-budding tissue process occupying the pharyngeal and supraglottic space, allowing air passage through the glottis, but causing complete supraglottic obstruction during inspiration. The patient responded well to bronchodilators and nebulized inhaled corticosteroids, showing slight improvement in respiratory function. The following day, the patient was taken to the operating room for tracheostomy tube extraction under fibroscopic guidance via the tracheostomy stoma. Preliminary dilation of the existing tracheostomy stoma was performed to permit the introduction of a size 7 cuffed tracheostomy tube, which was used to guide ventilation before anesthesia. Spontaneous ventilation using ketamine was employed, assisted by bronchoscopic guidance through the tracheostomy stoma to extract the migrated tube from the left mainstem bronchus ( Figures 4, 5); **(Video 1,2) (Extended data)**; ( Figure 6). A cuffed tracheostomy tube was then carefully repositioned to restore ventilatory support until the patient regained consciousness. A permanent tracheostomy will be performed at a later stage.

Post-procedure, the patient demonstrated significant improvement in oxygenation and respiratory effort. A follow-up chest radiograph confirmed improved aeration of the right lung. The patient was closely monitored for complications, including bronchial trauma, pneumonia, and pacemaker function. Cardiac telemetry did not show any arrhythmias related to hypoxia. The patient was discharged with strict follow-up and instructions for tracheostomy care to prevent recurrence.

## Discussion

Tracheostomy tubes bypass upper airway obstructions but may migrate—particularly into the left main bronchus—leading to airflow obstruction, pneumonia, and mechanical injury. The anatomy of the left bronchus renders it more susceptible to tube migration and subsequent complications, with risks increasing due to prolonged placement and inadequate monitoring. ^5^ Acute upper airway obstruction (UAO) requires urgent evaluation. Causes include infections, trauma, and mechanical blockages. Management strategies vary and may involve corticosteroids (e.g., dexamethasone for croup) and intubation in severe cases. ^6^ Tracheostomy is essential for securing the airway in patients with laryngeal and supraglottic cancers, especially when significant tumor-related narrowing is present, serving both emergency and perioperative roles, as demonstrated in our patient. ^7^ However, alternative minimally invasive methods are being explored. ^6^ Endoscopic techniques—such as laser-assisted surgery and flexible bronchoscopy—are used for foreign body removal and tumor debulking, thereby reducing complications (e.g., infections) and allowing real-time airway assessment and histopathological analysis. ^8^ When these noninvasive methods fail, tracheostomy becomes necessary. Notably, percutaneous tracheostomy performed under flexible bronchoscopy guidance is associated with fewer complications and faster recovery compared to surgical tracheostomy. ^9^

The diagnosis and management of post-tracheostomy migration (PTM) primarily rely on a thorough clinical assessment and review of the patient’s history. Stridor, respiratory distress, and airway obstruction are hallmark signs; however, their nonspecific nature often makes early recognition challenging. Given the significant overlap of symptoms—such as dyspnea, stridor, cough, and respiratory distress—with other respiratory conditions (e.g., pneumonia, bronchospasm, or upper airway obstruction), a high index of suspicion is required to promptly identify PTM and prevent life-threatening complications. Accurately localizing the tracheostomy tube remains challenging, particularly in critically ill patients where malposition or migration may not be evident on standard radiographic imaging. In contrast, bronchoscopy remains the gold standard for confirmation, although its availability may be limited in emergency settings. Consequently, emerging diagnostic techniques such as lung lavage assessment and ultrasound have been proposed as adjunct tools to enhance diagnostic accuracy and expedite intervention in high-risk patients. ^10^ In our patient, an urgent bronchoscopy was performed to validate tube positioning, reinforcing the critical role of direct visualization in PTM diagnosis. The integration of advanced imaging modalities, such as computed tomography (CT) and bronchoscopy, along with a multidisciplinary approach involving anesthesiologists, pulmonologists, and otolaryngologists, is crucial for accurately diagnosing tracheostomy tube migration. This comprehensive strategy enhances early detection, facilitates timely intervention, and ultimately improves patient outcomes by reducing complications associated with misplacement or delayed management. ^10^ In patients with additional comorbidities, such as cardiovascular disease requiring pacemakers, airway compromise can lead to hemodynamic instability. ^7^

Tracheostomy complications are common, with over 50% of patients experiencing issues such as decannulation and airway obstruction. These complications have been extensively studied in pediatric patients—especially those with congenital heart disease, bronchopulmonary dysplasia, and gastroesophageal reflux—who face higher risks of complications and mortality. ^11^ Common complications in children include accidental decannulation, tube obstruction, and increased mortality rates, particularly among neonates and infants with underlying respiratory disorders. ^2, 12^ Pediatric patients, or those with shortened necks, are particularly vulnerable to tracheostomy tube migration, especially during periods of agitation, as neck movement can contribute to tube dislodgment by altering its positioning. Additionally, prolonged antibiotic administration may compromise tissue integrity by disrupting normal wound healing and increasing susceptibility to infection, further elevating the risk of tube migration. ^12^ A thorough understanding of these risk factors is essential for mitigating serious complications. ^10^

Parida et al., ^2^ studied pediatric tracheostomy tube fractures and migration, identifying key factors that contribute to these issues. Fractures typically occur at the junction of the inner tube and the cervical plate, with fragments often migrating into the trachea or bronchus. Factors such as irregular follow-up, chronic coughing, inadequate care, and non-durable tubes were identified. Notably, in this study, 81.8% of pediatric patients exhibited a narrow stoma and granulation tissue, which significantly complicated the management of airway patency and tube stability. These issues pose a serious risk of respiratory obstruction in children, necessitating prompt interventions—such as rigid bronchoscopy—to remove the tube and avert life-threatening situations. Pediatric patients are especially at risk for complications; one study noted an 8% complication rate in tracheostomies, mainly among younger children. Key predictive factors include bronchopulmonary dysplasia, heart disease, and gastroesophageal reflux, with complications leading to longer hospital stays and more readmissions. ^13^

Cuestas et al., ^14^ reported a rare case of a tracheostomy tube fracture in a child leading to acute respiratory distress. They emphasize the need for regular follow-up and timely tube replacement, as well as educating patients and caregivers on proper care. Managing tube fractures usually involves rigid bronchoscopy, with early intervention being crucial to prevent severe airway obstruction. Furthermore, a prospective study by Nyanzi and Atwine in Uganda highlighted the significance of standardized protocols and multidisciplinary care in low-resource settings, noting tube obstruction as a common early complication in pediatric and high-risk patients. This risk can be mitigated through proper training in tracheostomy care—including correct tube placement, suctioning techniques, infection prevention, and early recognition of complications. Adherence to established guidelines, such as those for routine stoma care, tube changes, and emergency airway management, is essential for reducing complications and improving patient outcomes. ^3^ In addition, a study by Cheung and Napolitano, ^1^ reviewed tracheostomy procedures, examining epidemiology, indications, and associated risks. They found that although complications occurred in only about 10% of cases, they could be severe, including infections, bleeding, and airway obstructions. The timing and technique of the procedure—such as percutaneous versus open surgical tracheostomy—significantly influenced the incidence of complications, and comorbidities such as obesity and coagulopathy further increased the risk of adverse outcomes.

In contrast, adult studies have highlighted higher complication rates in older patients with comorbidities such as cardiac and hepatic diseases. Obstructive sleep apnea (OSA) has been identified as a significant predictor of post-tracheostomy complications in adults, whereas conditions like chronic obstructive pulmonary disease (COPD) and asthma show weaker correlations. ^7, 15^ These risk factors align with our patient’s history, as he had COPD and chronic ischemic heart disease complicated by arrhythmia that was resolved with pacemaker implantation. In a retrospective study by Murray et al., ^4^ reviewing tracheostomy complications from 2011 to 2018 in 697 patients, approximately 10% experienced complications such as bleeding, infections, and tracheal stenosis. Patients with severe comorbidities and challenging tracheostomy tube placements faced a higher risk of serious complications, including tube obstruction, displacement, and respiratory distress. These findings emphasize the importance of meticulous surgical planning and vigilant postoperative care to manage complications and prevent life-threatening outcomes, which closely aligns with our case.

Similarly, Higashino et al., ^13^ reported the case of a 65-year-old man whose metallic tracheostomy tube fractured and migrated into the left main bronchus 18 months after implantation, leading to acute respiratory distress. The condition was successfully managed with rigid bronchoscopy—a scenario comparable to our case. This highlights the necessity for regular tube replacement, as long-term use of metallic tubes can lead to corrosion and fracture. A Finnish study by Ruohoalho, Xin, and Bäck, ^8^ involving 255 patients found that while complications were infrequent, they could still be significant; 22% of patients experienced complications, with pneumonia and accidental decannulation being the most common. These findings underscore the importance of careful management of difficult airways, particularly in cases requiring ear, nose, and throat (ENT) surgical intervention.

Preventive measures and increased monitoring of high-risk patients are essential strategies to minimize postoperative complications and improve outcomes. These measures should include a careful evaluation of risk factors and methods for securing the tube, especially in patients with cerclage sutures. Selecting the appropriate tube—cuffed for adults and uncuffed for children—is crucial, and using internal cannulas facilitates maintenance. Routine monitoring, timely tube replacement, and patient education regarding signs of displacement are imperative for risk reduction. ^16^ Given that tracheostomy tubes can remain indwelling for long durations, proper sizing is necessary to prevent injury or migration; oversized tubes risk injuring the trachea, while undersized tubes may be prone to dislodgment. ^17^ Correct tube positioning is vital, as excessive migration can result from overly low placement or anatomical distortions.

In addition, several stabilization strategies—including stay sutures—may be employed to avert dislodgment. Prolonged ventilation correlates with an increased likelihood of complications, underscoring the need for meticulous management. Understanding the variables that influence tracheostomy tube migration—such as advanced age and increased intra-abdominal pressure—can help inform preventive measures. Research by Mussa et al., ^15^ supports restricting tube removal procedures to healthcare professionals, as this ensures proper assessment and minimizes the risk of complications. Early recognition of migration risks may alleviate respiratory complications and enhance patient outcomes. Furthermore, the rising incidence of tracheostomy placements in the aging population emphasizes the need for specialized interventions and structured clinical protocols. Patient education regarding tracheostomy tube management and care is also paramount for preventing complications and improving long-term outcomes. ^15^

Advancements in telemedicine hold promise for improving management and follow-up care; however, effective care coordination is essential for managing the various comorbidities—such as COPD, neuromuscular disorders, and coagulation abnormalities—that can complicate tracheostomy care. ^18^ Thorough preoperative evaluations, including airway assessments, imaging studies (e.g., CT scans to assess tracheal anatomy), and multidisciplinary consultations with anesthesiologists, pulmonologists, and otolaryngologists, can help minimize migration risks. Institutional support is critical for ensuring timely access to specialized consultations, post-placement monitoring, and emergency intervention protocols to optimize patient outcomes. A comprehensive approach to management is imperative for addressing complications, and extensive studies are needed to evaluate the efficacy of various strategies. Effective management also involves securing informed consent from the patient or their legal guardian—particularly when tracheostomy tube placement or replacement is planned—to ensure that the procedure, its potential risks, and alternative options are fully understood, thereby reinforcing shared decision-making and ethical medical practice. ^19^

Continuous monitoring and the option for surgical intervention are essential to prevent and manage complications. Ng et al., ^19^ emphasize the importance of careful preparation when replacing tracheostomy tubes due to risks such as infections and airway obstructions. They highlight the necessity of using sterile equipment, ensuring mucosal hydration, and conducting immediate postoperative observation to mitigate these risks. Furthermore, proper training of medical personnel is crucial for minimizing adverse outcomes, and ongoing education for healthcare providers is essential to maintain high standards of care during tracheostomy tube changes. A study by Cramer, Graboyes, and Brenner, ^20^ on tracheostomy-related mortality in the U.S. (2007–2016) found a higher incidence of fatal complications in African American children, underscoring the need for targeted interventions to reduce this mortality risk. The authors advocated for improved care protocols and early management strategies to lower the tracheostomy-related mortality rate, emphasizing an individualized approach for high-risk groups.

Managing complications such as tube migration requires regular inspections, with chest radiography and bronchoscopy serving as essential diagnostic tools for detecting and addressing tube misplacement and related airway complications. ^20^

The Clinical Consensus Statement (CCS), developed by Mussa et al. (2021), ^15^ outlines standardized protocols for tracheostomy care with a strong focus on patient and caregiver education, emergency preparedness, and decannulation readiness. These guidelines aim to enhance patient safety and improve long-term outcomes by ensuring proper tracheostomy management.

Guidelines proposed by Rovira et al. (2021), ^21^ emphasize early tracheostomy intervention, recommending placement within 10 days of intubation to reduce the risk of ventilator-associated complications. They also advise regular tracheostomy tube replacement every three to six months to prevent issues such as granulation tissue formation and tube obstruction. However, this recommended practice was not followed in our patient, highlighting the importance of strict adherence to established protocols to minimize risks.

Studies comparing surgical and percutaneous tracheostomy approaches suggest that percutaneous tracheostomy performed under bronchoscopic guidance is associated with fewer complications compared to the open surgical technique. ^9^

A multidisciplinary approach involving intensivists, respiratory therapists, and ENT specialists is recommended to optimize patient care. Regular monitoring through bronchoscopy and radiography is advised for the early detection and management of complications. Additionally, education and training programs for healthcare providers are essential to address knowledge gaps in areas such as cuff pressure management, blockage handling, and stomal infection identification. ^18, 21^ Despite its effectiveness, tracheostomy carries risks—such as hemorrhage—and has a 10% complication rate within 90 days, emphasizing the importance of careful patient selection and management. ^18, 21^

## Conclusion

Tracheostomy is a life-saving intervention for upper airway obstruction but presents challenges that demand careful patient selection, thorough preoperative assessment, and strict adherence to standardized protocols. This study highlights the risks of tracheostomy tube migration and stresses the need for early detection, timely intervention, and a multidisciplinary approach to prevent life-threatening events. Regular clinical and radiographic evaluations of tube positioning are vital, especially for patients with altered airway anatomy or implanted devices. Bronchoscopy remains the gold standard for confirming and correcting mispositioned tubes to maintain airway patency. Proactive monitoring and evidence-based management are essential for optimizing patient care, reducing morbidity, and enhancing quality of life.

## Author contributions

**Said Khallikane:** Conceptualization, Investigation, Writing–original draft. **Amine Bentaher:** Assistance in obtaining references, and Editing. **Monsif Salek:** Conceptualization, Investigation, Writing–original draft **.**. **Ayoub Belhadj:** Investigation, Validation, Writing–review and editing. **Younes Aissaoui:** Investigation, Validation, and Supervision.

## Consent to publish

Written informed consent for publication of his clinical details, clinical images and clinical videos was obtained from the patient.

## Data Availability

No data are associated with this article. Dataset Figshare 1: Peroperative bronshoscopy showing the tracheostomy tube within the left main bronchus, causing an inflammatory reaction and conforming to the bronchial wall. https://doi.org/10.6084/m9.figshare.28635305.v1 ^22^ This dataset contains following Media file
- Bronchoscopy 1.mp4 Dataset 2 Figshare 2: Peroperative bronchoscopy showing the Intraoperative bronchoscopy performed through the tracheostomy orifice to facilitate the forceps extraction of a migrated tracheostomy tube lodged in the left main bronchus. https://doi.org/10.6084/m9.figshare.28635308.v1. ^23^ This dataset contains following Media file
- Bronchoscopy 2.mp4 Data are available under the terms of the Creative Commons Zero “No rights reserved” data waiver (CC0 1.0 Public domain dedication).

## References

[1] Cheung NH Napolitano LM : Tracheostomy: Epidemiology, Indications, Timing, Technique, and Outcomes. *Respir. Care.* 2014;59(6):895–919. 10.4187/respcare.02971 24891198

[2] Parida P-K Raja K Alexander A : Factors Associated with Fracture and Migration of Tracheostomy Tube into Trachea in Children: A Case Series. *PubMed.* 2020;32(113):379–383. 10.22038/ijorl.2020.44797.2473 33282786 PMC7701484

[3] Nyanzi DJ Atwine D Kamoga R : Tracheostomy-related indications, early complications and their predictors among patients in low resource settings: a prospective cohort study in the pre-COVID-19 era. *BMC Surg.* 2023;23(1):59. 10.1186/s12893-023-01960-5 36934224 PMC10024521

[4] Murray M Shen C Massey B : Retrospective analysis of post-tracheostomy complications. *Am. J. Otolaryngol.* 2021;43(2):103350. 10.1016/j.amjoto.2021.103350 34974381

[5] Eskander A Almeida JR de Irish JC : Acute Upper Airway Obstruction. *N. Engl. J. Med.* 2019;381(20):1940–1949. 10.1056/nejmra1811697 31722154

[6] Choudhury N Perkins V Amer I : Endoscopic airway management of acute upper airway obstruction. *Eur. Arch. Otorrinolaringol.* 2013;271(5):1191–1197. 10.1007/s00405-013-2618-6 23836440

[7] Weir CJ : Stoelting’s Anesthesia and Co-existing Disease. *Br. J. Anaesth.* 2013;110(5):858. 10.1093/bja/aet081

[8] Ruohoalho J Xin G Bäck L : Tracheostomy complications in otorhinolaryngology are rare despite the critical airway. *Eur. Arch. Otorrinolaringol.* 2021;278:4519–4523. 10.1007/s00405-021-06707-7 33656585 PMC8486710

[9] Stawicki S Cipriano A Mao M : An overview of complications associated with open and percutaneous tracheostomy procedures. *Int. J. Crit. Illn. Inj. Sci.* 2015;5(3):179–188. 10.4103/2229-5151.164994 26557488 PMC4613417

[10] Guedes F Branquinho MV Sousa AC : Central airway obstruction: is it time to move forward? *BMC Pulm. Med.* 2022;22(1):68. 10.1186/s12890-022-01862-x 35183132 PMC8858525

[11] Singh A Zubair A : Pediatric Tracheostomy. *PubMed.* 2023. [Accessed 12 Dec. 2023]. Reference Source

[12] Morrison JM Hassan A Kysh L : Diagnosis, management, and outcomes of pediatric tracheostomy-associated infections: A scoping review. *Pediatr. Pulmonol.* 2022;57(5):1145–1156. 10.1002/ppul.25873 35229491 PMC9313552

[13] Higashino M Saito K Tsukahara K : Tracheostomy in otorhinolaryngology education and training programs: A Japanese nationwide survey. *Auris Nasus Larynx.* 2023;51(1):69–75. 10.1016/j.anl.2023.08.003 37563043

[14] Rotura de la cánula de traqueostomía: una causa rara de dificultad respiratoria en el niño traqueotomizado. Caso clínico. *Arch. Argent. Pediatr.* 2015;113(6):e353–e356. 10.5546/aap.2015.e353 26593816

[15] Mussa CC Gomaa D Rowley DD : AARC Clinical Practice Guideline: Management of Adult Patients with Tracheostomy in the Acute Care Setting. *Respir. Care.* 2020;66(1):156–169. 10.4187/respcare.08206 32962998

[16] Morris LL Whitmer A McIntosh E : Tracheostomy Care and Complications in the Intensive Care Unit. *Crit. Care Nurse.* 2013;33(5):18–30. 10.4037/ccn2013518 24085825

[17] Lavo JP Ludlow D Morgan M : Predicting feeding tube and tracheotomy dependence in laryngeal cancer patients. *Acta Otolaryngol.* 2017;137(3):326–330. 10.1080/00016489.2016.1245864 27780384

[18] Ezeamii VC Okobi OE Wambai-Sani H : Revolutionizing Healthcare: How Telemedicine Is Improving Patient Outcomes and Expanding Access to Care. *Cureus.* 2024;16(7):e63881. 10.7759/cureus.63881 39099901 PMC11298029

[19] Ng J Hamrang-Yousefi S Hohman MH : Tracheostomy Tube Change. *StatPearls.* 2024. Reference Source 32310379

[20] Cramer JD Graboyes EM Brenner MJ : Mortality associated with tracheostomy complications in the United States: 2007-2016. *Laryngoscope.* 2018;129(3):619–626. 10.1002/lary.27500 30450581

[21] Rovira A Dawson D Walker A : Tracheostomy care and decannulation during the COVID-19 pandemic. A multidisciplinary clinical practice guideline. *Eur. Arch. Otorrinolaringol.* 2020;278:313–321. 10.1007/s00405-020-06126-0 32556788 PMC7299456

[22] Khallikane S : Peroperative bronshoscopy showing the tracheostomy tube within the left main bronchus, causing an inflammatory reaction and conforming to the bronchial wall. *figshare. Media.* 2025. 10.6084/m9.figshare.28635305.v1

[23] Khallikane S : Peroperative bronchoscopy showing the Intraoperative bronchoscopy performed through the tracheostomy orifice to facilitate the forceps extraction of a migrated tracheostomy tube lodged in the left main bronchus. *figshare. Media.* 2025. 10.6084/m9.figshare.28635308.v1

