# Supplementary material for: Case Report- Prevention strategies for permanent tracheostomy tube migration into the left main bronchus in tracheostomized patients
Source: F1000Res. 2025 Apr 1;14:372. [Version 1] doi: 10.12688/f1000research.163217.1 (PMC12079324; doi:10.12688/f1000research.163217.1)
Supplement: Peer review history

# Reviewer report: Reviewer response for version 1

**DOI:** 10.5256/f1000research.179527.r379803

**Published:** 2025-05-10

**Authors:** Noureddine Atmani

**Affiliations:** 1 Mohamed V University of Rabat, Rabat, Morocco

This article presents a rare and potentially life-threatening case of **tracheostomy tube migration** in a 66-year-old man with multiple chronic conditions, including **COPD**, **ischemic cardiomyopathy with a pacemaker**, and **supraglottic squamous cell carcinoma**. The tracheostomy had been performed due to upper airway obstruction from the cancer.

 The patient presented to the emergency department with **respiratory distress** and was found to have a **dislodged tracheostomy tube**, which imaging and bronchoscopy confirmed had migrated into the **left main bronchus**, causing partial airway obstruction. Emergency **bronchoscopic removal** of the tube was performed under **spontaneous ventilation and light anesthesia**, and a new cuffed tracheostomy tube was correctly repositioned.

 The discussion emphasizes that **tracheostomy tube migration**, though rare in adults, can have serious outcomes. Bronchoscopy remains the **gold standard** for diagnosis and correction. The article reviews similar cases in the literature, particularly in pediatric populations, and identifies common risk factors such as **poor tube maintenance, prolonged use, comorbidities**, and **lack of routine monitoring**.

 Preventive strategies include:

- Proper **tube selection and sizing**
- Regular **tube replacement**
- **Education** for caregivers and healthcare providers
- Use of **bronchoscopic guidance** during procedures
- A **multidisciplinary approach** involving ENT, pulmonology, anesthesia, and nursing

The **conclusion** stresses the importance of early recognition, patient-specific care, adherence to standardized protocols, and close monitoring to prevent severe complications and improve patient outcomes.

Are enough details provided of any physical examination and diagnostic tests, treatment given and outcomes?

Yes

Is the case presented with sufficient detail to be useful for other practitioners?

Yes

Is sufficient discussion included of the importance of the findings and their relevance to future understanding of disease processes, diagnosis or treatment?

Yes

Is the background of the case’s history and progression described in sufficient detail?

Yes

Reviewer Expertise:

cardiac surgery

I confirm that I have read this submission and believe that I have an appropriate level of expertise to confirm that it is of an acceptable scientific standard.

# Response

**Published:** 2025-05-10

**Authors:** said khallikane

thank you for you for yout throughful review, We appreciate your positive assessment and we are pleased that the case report was found sufficient to be sufficientlu detailed and relevant for clinical practice. We believe that the current content effectively adressses the key aspects of diagnosis, management and broader implications without requiring futher amendments.....

# Reviewer report: Reviewer response for version 1

**DOI:** 10.5256/f1000research.179527.r379799

**Published:** 2025-04-30

**Authors:** Rachid Benchanna

**Affiliations:** 1 Service de pneumologie, HMA, Marrakech, Morocco

In observation, the quantification of the degree of smoking must be clearly specified: you are talking about 35 years of tobacco while you are objectifying two packs per year.

 For the treatment of COPD you must specify the group to which the patient belongs according to the GOLD 2025 recommendations or at least if it is a frequent exacerbator specify it: say that it is a frequent exacerbator, and then say that it is on inhaled triple therapy: LABA, LLAMA, ICS.

 You must specify the type of fibroscopy performed: apparently it is a flexible fibroscopy initially for diagnostic purposes. Was the extraction of the cannula performed by a flexible or rigid endoscopy?

 In the discussion part: the right stem bronchus is normally more vulnerable to the migration of foreign bodies and it is not the other way around as you mentioned, because of its more vertical angle and its larger diameter.

Are enough details provided of any physical examination and diagnostic tests, treatment given and outcomes?

Partly

Is the case presented with sufficient detail to be useful for other practitioners?

Yes

Is sufficient discussion included of the importance of the findings and their relevance to future understanding of disease processes, diagnosis or treatment?

Yes

Is the background of the case’s history and progression described in sufficient detail?

Partly

Reviewer Expertise:

pulmonologist

I confirm that I have read this submission and believe that I have an appropriate level of expertise to confirm that it is of an acceptable scientific standard.

# Response

**Published:** 2025-04-30

**Authors:** said khallikane

We thank the reviewer for their insightful remarks and appreciate the opportunity to clarify several points raised:

1. **Smoking Quantification:**
  The reference to “35 years of tobacco” was intended to convey the long-term exposure rather than a precise pack-year calculation. While the estimated consumption may appear modest numerically, our aim was to emphasize duration as a contributing factor in the clinical profile.
2. **COPD Classification:**
  We note the importance of GOLD classification. While a detailed subgrouping was not explicitly included, we chose to describe the patient as a “frequent exacerbator” receiving triple inhaled therapy (ICS, LABA, LAMA), which we felt sufficiently reflected the severity and management approach in clinical practice.
3. **Bronchoscopy Technique:**
  Thank you for this important point. The case involved flexible bronchoscopy initially for diagnostic purposes, as is typical in our setting. The removal of the foreign body was performed via endoscopic guidance. While the exact instrumentation was not specified, the focus remained on the outcome and clinical significance.
4. **Discussion on Bronchial Anatomy:**
  We acknowledge the reviewer’s comment and understand that anatomically, the right main bronchus is typically more predisposed to foreign body migration. Our phrasing was intended to reflect the findings in this particular case rather than general anatomical patterns.
5. **Case History and Progression:**
  We believe the case’s progression is presented with sufficient detail to allow the reader to follow the diagnostic reasoning, clinical decisions, and therapeutic approach, while maintaining conciseness and readability.

We are grateful for the reviewer’s thoughtful engagement and hope that this clarification supports the intent and structure of the submitted work.
